# Inflammation vs. multiple AVN sites: a nuclear medicine evaluation in systemic allergic-immune syndrome – a case report

**DOI:** 10.1097/MS9.0000000000005554

**Published:** 2026-08-25

**Authors:** Ayan Dhiman, Roshan Karna, Mehul Taggar, Shamim Ahmed Shamim

**Affiliations:** a Department of Nuclear Medicine, All India Institute of Medical Sciences (AIIMS), New Delhi, India; b Outpatient Department, Lele Primary Health Centre, Lalitpur, Bagmati, Nepal

**Keywords:** 99mTc-MDP bone scintigraphy, avascular necrosis, case report, corticosteroid, migratory polyarthralgia, MRI, SPECT/CT

## Abstract

**Introduction:**

Migratory polyarthralgia often indicates inflammatory or autoimmune pathology. In patients with concomitant allergic diathesis and immune dysregulation, diagnosis becomes particularly challenging, as conventional laboratory and serological evaluations frequently remain inconclusive. Nuclear medicine, particularly three-phase 99mTc-MDP bone scintigraphy with SPECT/CT, offers functional insights that overcome these limitations by directly visualizing bone metabolic activity, independent of serological status.

**Case presentation:**

A 27-year-old woman with bronchial asthma and allergic bronchopulmonary aspergillosis (ABPA) presented with an 11-month history of migratory polyarthralgia. Her background included recurrent anaphylaxis, multiple prior viral infections, and significant systemic corticosteroid exposure (intravenous methylprednisolone 40 mg/day for 10 days, followed by oral methylprednisolone 30 mg three times daily, with subsequent tapering over a total course from September to November 2024), resulting in cushingoid features. Three-phase 99mTc-MDP bone scintigraphy demonstrated increased tracer uptake across multiple joints, consistent with inflammatory arthropathy, and SPECT/CT correlation revealed multifocal avascular necrosis (AVN) and subclinical synovitis in the bilateral femoral heads, elbows, wrists, knees, and ankles. MRI of the right knee and both hips provided anatomical confirmation.

**Clinical discussion:**

This case illustrates the diagnostic utility of bone scintigraphy in immune-allergic overlap syndromes in which conventional investigations are inconclusive. SPECT/CT enables the simultaneous delineation of inflammatory and ischemic etiologies, directly guiding therapeutic decision-making, including bisphosphonate therapy and joint-protective rehabilitation.

**Conclusion:**

Three-phase 99mTc-MDP bone scintigraphy with SPECT/CT serves as a crucial imaging modality in evaluating complex polyarthralgia. These findings enabled timely therapeutic intervention, including reassessment of corticosteroid use, institution of bisphosphonate therapy (zoledronic acid), and joint-protective rehabilitation, illustrating the direct clinical impact of advanced nuclear medicine imaging. The persistence of symptoms despite treatment highlights the refractory nature of multifocal corticosteroid-induced AVN and the need for continued multidisciplinary follow-up.

## Introduction and importance

Polyarthralgia with migratory patterns often suggests inflammatory, autoimmune, or reactive etiologies. When compounded by immune dysregulation and allergic diathesis, the diagnostic process becomes complex. Standard rheumatologic serological evaluations, including rheumatoid factor (RF), anti-cyclic citrullinated peptide antibodies (anti-CCP), antinuclear antibodies (ANA), and acute-phase reactants (ESR, CRP), frequently yield inconclusive results in patients with immune-allergic overlap syndromes. In such patients, elevated immunoglobulin E (IgE), peripheral eosinophilia, and polyclonal immune activation produce a non-specific inflammatory milieu that confounds serological interpretation. Compounding this, systemic corticosteroid therapy – a cornerstone of management for allergic and autoimmune diseases – suppresses inflammatory markers while simultaneously predisposing patients to AVN through disruption of lipid metabolism, increased intraosseous pressure, and microvascular compromise^[^1^]^. This dual effect renders conventional work-up particularly unreliable in characterizing the underlying joint pathology. Three-phase 99mTc-MDP bone scintigraphy with SPECT/CT overcomes these limitations by directly visualizing bone metabolic activity and perfusion changes, independent of serological status, enabling simultaneous detection of both active synovitis and ischemic changes.

HIGHLIGHTS

- Migratory polyarthralgia poses significant diagnostic challenges.
- 99mTc-MDP bone scintigraphy with SPECT/CT aided in the detection of subclinical inflammation.
- An MRI confirmed early avascular necrosis in a steroid-exposed patient.
- Multimodal imaging-guided therapeutic decisions in a complex immune background.
- Integration of nuclear and anatomic imaging improved diagnostic confidence.

This case report has been prepared in accordance with the updated TITAN 2025 checklist for transparent and comprehensive reporting of medical cases^[^2^]^.

## Case presentation

### Medical history

A 27-year-old female presented to the Department of Nuclear Medicine with a 11-month history of migratory polyarthralgia involving both large and small joints. Pain was exacerbated by physical activity and stress and relieved by rest and medication. The patient had a known history of bronchial asthma for 5 years and was diagnosed with Allergic Bronchopulmonary Aspergillosis (ABPA) during a severe exacerbation in September 2024. At that time, she required CPAP support and was noted to have tachycardia, respiratory alkalosis, hypotension, and leukopenia. She received intravenous methylprednisolone 40 mg/day for 10 days during the acute ABPA exacerbation, followed by oral prednisolone 30 mg three times daily (90 mg/day), with subsequent dose tapering. The total corticosteroid course extended from September 2024 to November 2024. Cushingoid features developed within 10 days of initiating therapy, reflecting the high cumulative steroid burden. Prior hospitalizations for anaphylaxis and viral illnesses had also necessitated short courses of systemic corticosteroids over the preceding years, further adding to the cumulative exposure.

She reported multiple episodes of anaphylaxis to unknown environmental and dietary allergens (e.g., seafood, honey, and strawberries), requiring subcutaneous adrenaline administration, with – the most recent episode occurring in June 2024. She had several hospital admissions during her MBBS (2017–2025), including for dengue shock syndrome, dengue hemorrhagic fever, swine flu, COVID-19, and dengue fever post-leukopenia.

### Surgical history

Past surgical history includes congenital epigastric hernia repair (2008) and laparoscopic appendectomy (2017), both of which were uneventful.

### Family history

Family history revealed an allergic diathesis in her father, brother (bronchial asthma), and sister (urticaria, unknown allergies), indicating a strong hereditary atopic background.

### Psychosocial history

A psychosocial trigger was identified: the sudden death of her younger brother in a road traffic accident, following which her systemic symptoms flared.

### Imaging findings

**Figure 1.**
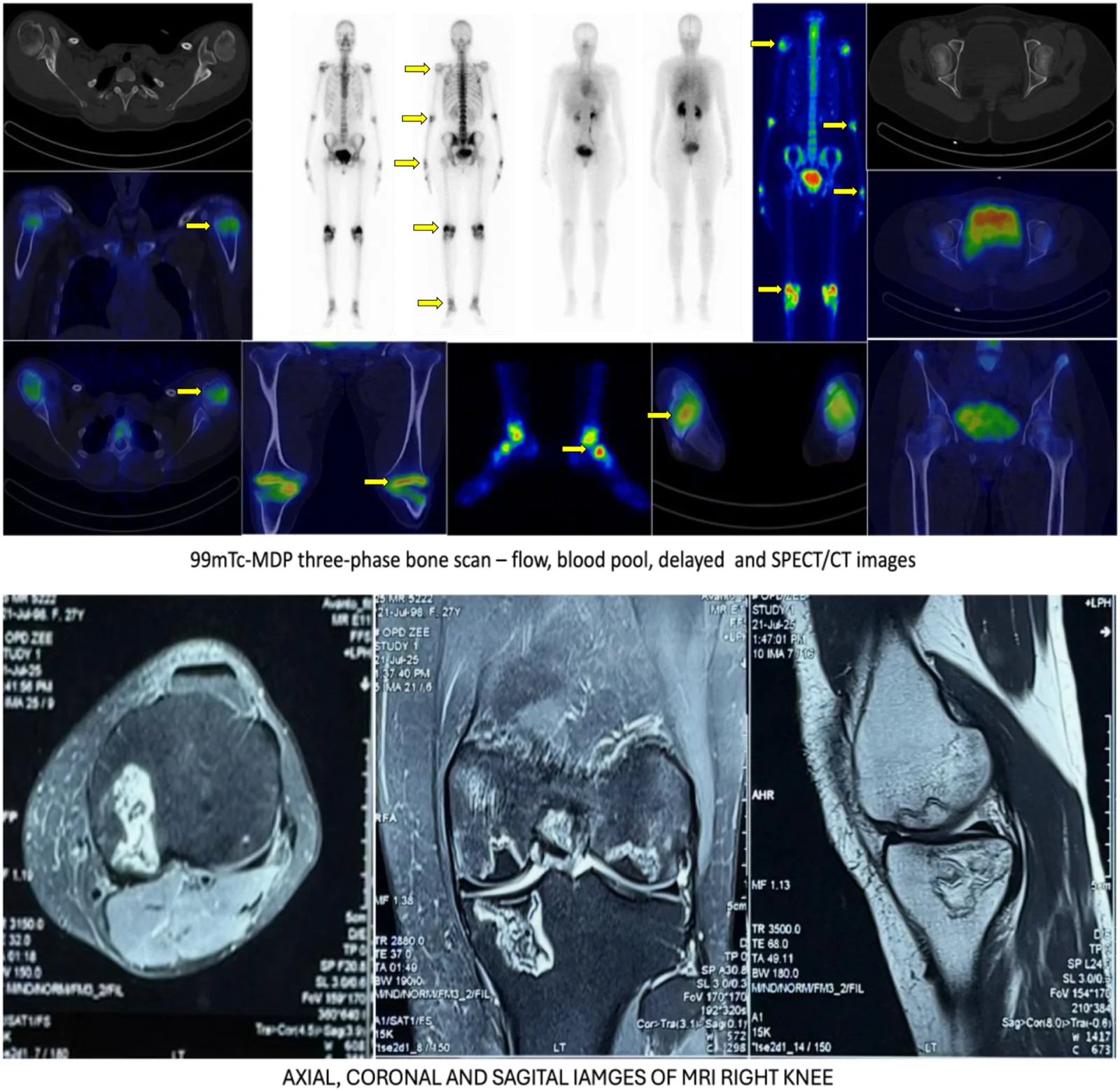
Imaging findings.

A three-phase 99mTc-MDP bone scan was performed to evaluate inflammatory joint involvement versus multiple AVN sites. The flow images showed no increased radiotracer activity in the bilateral shoulder regions, and the blood pool images showed increased radiotracer activity in the bilateral knee and ankle regions. Delayed planar images demonstrated increased radiotracer uptake in the bilateral shoulders, elbows, wrists, hips, knees, and ankles, as well as in the small joints of the hands and feet, consistent with inflammatory arthropathy. SPECT/CT provided anatomical correlation, particularly in the bilateral radial heads, olecranon processes of the ulnae, femoral heads, distal femora, proximal tibiae, and tali, supporting findings of avascular necrotic changes, and demonstrated increased tracer activity in the bilateral wrists, small joints of both hands, bilateral ankles, and bilateral talonavicular joints, supporting findings of subclinical joint inflammation. The findings are shown in Figure 1.

A three-phase 99mTc-MDP bone scan was performed to evaluate inflammatory joint involvement versus multiple AVN sites. The flow images show no increased radiotracer activity in the bilateral shoulder region, and the blood pool images show increased radiotracer activity in the bilateral knee and ankle regions. Delayed planar images demonstrated increased radiotracer uptake (yellow arrows) in the bilateral shoulders, elbows, wrists, hips, knees, and ankles, and the small joints of the hands and feet, consistent with inflammatory arthropathy. SPECT/CT provided anatomical correlation (yellow arrows), particularly in the bilateral radial heads, olecranon processes of the ulnae, femoral heads, distal femora and proximal tibiae, and the tali, supporting findings of avascular necrotic changes, and increased tracer activity in the bilateral wrists, the small joints of both hands, the bilateral ankles, and the bilateral talonavicular joints, supporting findings of subclinical joint inflammation. MRI of the right knee confirmed AVN changes in the distal femur and proximal tibia, consistent with the SPECT/CT findings, without evidence of subchondral collapse at the time of imaging.

Complementary MRI examinations of the right knee and both hips were performed for anatomical characterization. MRI of the right knee confirmed AVN changes in the distal femur and proximal tibia. MRI of both hips demonstrated AVN changes in both femoral heads, consistent with the SPECT/CT findings, without evidence of subchondral collapse at the time of imaging.

### Follow-up and clinical outcomes

Following the multimodal imaging workup, the patient was reviewed by a multidisciplinary team comprising nuclear medicine, rheumatology, orthopedics, and allergy/immunology specialists. Systemic corticosteroid use was minimized and steroid-sparing strategies were prioritized for management of the underlying allergic disease. Joint-protective physiotherapy was initiated. Orthopedic consultation was sought for the femoral head AVN, and in view of the imaging findings, an infusion of zoledronic acid (an intravenous bisphosphonate) was administered on 20 August 2025 with the aim of reducing bone resorption and potentially halting AVN progression.

The patient experienced initial symptomatic improvement following the zoledronic acid infusion. However, over approximately 2 months leading up to the current assessment (February 2026), her symptoms returned and progressively worsened, with bilateral lower-limb arthralgia now significantly restricting joint movement and daily activities. All details are summarized in Table 1. At the time of writing, the patient is not receiving any active pharmacologic management. No further bisphosphonate infusion or surgical intervention has been scheduled, and long-term follow-up with repeat imaging is warranted to reassess disease status and guide further therapeutic decisions.

**Table 1:** Summary of key clinical events, steroid exposure, imaging timeline, and follow-up.

Date/Period	Event/Category	Details
2008	Surgical	Congenital epigastric hernia repair
2017	Surgical	Laparoscopic appendectomy
2017–2025	Recurrent hospitalizations	Dengue fever (×3), dengue shock syndrome, dengue hemorrhagic fever, swine flu, COVID-19; short steroid courses during each admission
June 2024	Anaphylaxis	Most recent episode; subcutaneous adrenaline required
September 2024	ABPA exacerbation	CPAP support; IV methylprednisolone 40 mg/day × 10 days → oral prednisolone 30 mg TDS → taper; total course: Sep–Nov 2024; cushingoid features at day 10
2024 (around Sep–Oct)	Psychosocial stressor	Sudden death of younger brother (road traffic accident); systemic flare followed
11 months pre-presentation	Symptom onset	Migratory polyarthralgia: bilateral large and small joints; worsened with activity, relieved by rest and analgesia
At presentation	Bone scintigraphy (3-phase)	Increased tracer uptake: bilateral shoulder, elbow, wrist, hip, knee, ankle, small joints of hands and feet – inflammatory arthropathy pattern. Flow: no increased activity bilateral shoulders. Blood pool: increased activity bilateral knees and ankles.
At presentation	SPECT/CT	AVN changes: bilateral radial heads, olecranon processes, bilateral femoral heads, bilateral distal femur/proximal tibia, talus. Subclinical synovitis: bilateral wrists, small hand joints, bilateral ankles, bilateral talonavicular joints.
At presentation	MRI	Right knee: AVN changes confirmed. Bilateral hips: AVN changes at femoral heads confirmed.
Post-imaging	Multidisciplinary review	Orthopedics, rheumatology, and allergy/immunology consultation; steroid-sparing strategy initiated; physiotherapy commenced
August 2025	Bisphosphonate therapy	Zoledronic acid infusion administered for AVN management
Aug 2025 (after infusion)	Initial response	Symptomatic improvement noted following zoledronic acid
Feb 2026 onwards (2 months before current assessment)	Symptom recurrence	Worsening arthralgia, progressive restriction of joint movements; bilateral lower limb mobility most affected
Current (April 2026)	Current status	No active pharmacological management being received; symptoms ongoing and significantly limiting mobility

## Clinical discussion

This case illustrates the diagnostic complexity of migratory polyarthralgia in the presence of systemic allergic and immune dysregulation. Despite an ambiguous clinical profile and nonspecific laboratory findings, nuclear medicine imaging provided objective, simultaneous evidence of both active synovitis and multifocal avascular necrosis – two pathologies requiring fundamentally different management strategies. Bone scintigraphy, especially when combined with SPECT/CT, serves as a sensitive modality for early detection of synovitis, for monitoring progression, and for guiding immunomodulatory and orthopedic treatment.

Published evidence supports the role of bone scintigraphy in complex arthropathy: Lee *et al* (2021) showed that quantitative bone scintigraphy strongly correlates with disease activity in rheumatoid arthritis, with approximately 79% sensitivity for detecting joint inflammation^[^3^]^. Wenger and Schirmer^[^4^]^ emphasized the growing role of nuclear imaging in early inflammatory arthropathies, advocating its use alongside serological markers. Al-Nahhas *et al*^[^5^]^ demonstrated the value of serial bone scans in monitoring subclinical synovitis and therapeutic response. Schmidkonz *et al*^[^6^]^ explored FAPI PET/CT for fibroblast activity in arthritis, with nearly 100% sensitivity, representing a potential future adjunct to MDP imaging.

## Comparison with previously reported cases

Corticosteroid-induced AVN is a well-documented entity, with published estimates suggesting systemic steroid use accounts for approximately 35% of non-traumatic AVN cases^[^1^]^, most commonly affecting the femoral head bilaterally. However, the simultaneous occurrence of multifocal AVN extending to non-weight-bearing sites – including the radial heads and olecranon processes – alongside active polyarticular synovitis, as documented in our patient, is far less frequently reported. Prior reports of ABPA patients on prolonged corticosteroid therapy have described femoral head AVN in relative isolation, but the concurrent presentation of recurrent immune-mediated polyanaphylaxis with multi-joint involvement of both large- and small joints represents an exceptionally complex and under-described clinical scenario. Furthermore, the partial and temporary response to bisphosphonate therapy followed by symptom recurrence observed in our patient reflects the challenging treatment landscape of multifocal corticosteroid-induced AVN, which lacks robust evidence-based therapeutic guidelines^[^7^]^. The utility of three-phase bone scintigraphy in simultaneously delineating both inflammatory and ischemic joint pathology in a single imaging session remains underutilized in immune-allergic populations, and our case contributes to the growing body of evidence supporting its clinical role in this context.

## Management implications

The multimodal imaging findings had direct and significant implications for clinical management. Detection of multifocal AVN in the bilateral femoral heads and other sites prompted urgent orthopedic consultation and a critical reassessment of the corticosteroid-prescribing strategy, favoring steroid-sparing immunomodulatory alternatives for the underlying allergic disease. The subclinical synovitis identified in the bilateral wrists, the small joints of the hands, the ankles, and the talonavicular joints guided the rheumatology team toward joint-protective measures, including physiotherapy and activity modification. Bisphosphonate therapy in the form of intravenous zoledronic acid was administered on 20 August 2025, providing initial symptom relief. However, the subsequent recurrence of symptoms and progressive mobility restriction despite this intervention underscore the refractory nature of established multifocal AVN and highlight the urgent need for ongoing multidisciplinary review, repeat functional imaging, and consideration of further therapeutic options, including surgical consultation for the most severely affected weight-bearing joints. Serial bone scintigraphy is recommended to objectively monitor disease trajectory and response to any future interventions.

## Limitations

This report carries inherent limitations common to single-case publications. First, findings from a single patient cannot be generalized to the broader population of patients with immune-allergic overlap syndromes; prospective studies are needed to validate these observations. Second, histopathological confirmation of AVN was not obtained; the diagnosis was established on the convergence of clinical risk factors (corticosteroid exposure and immune dysregulation) and multimodal imaging findings, which were deemed sufficient to guide management without the risk of invasive biopsy. Third, formal quantification of serological and inflammatory laboratory markers was not available, precluding correlation of functional imaging findings with biochemical disease activity. Fourth, the precise cumulative corticosteroid burden from multiple prior hospitalizations could not be fully quantified, limiting characterization of the dose-response relationship between steroid exposure and AVN. Finally, the patient is currently without active pharmacological management despite progressive symptoms, and longer follow-up with repeat imaging is required before definitive conclusions regarding disease trajectory can be drawn.

## Conclusion

Three-phase 99mTc-MDP bone scintigraphy with SPECT/CT serves as a critical and irreplaceable imaging modality in the evaluation of polyarthralgia in patients with complex immune and allergic backgrounds. By simultaneously characterizing both inflammatory and ischemic joint pathology, it enables differentiation between concurrent conditions that conventional workup cannot readily distinguish. In this case, functional imaging directly guided therapeutic decision-making, including steroid minimization and bisphosphonate therapy, while the partial and temporary treatment response to zoledronic acid, followed by symptom recurrence, underscores the refractory nature of multifocal corticosteroid-induced AVN. Continued multidisciplinary follow-up and repeat functional imaging remain essential in such patients. This case adds to the limited literature on the coexistence of immune-allergic polyanaphylaxis and multifocal AVN and highlights the need for early and comprehensive nuclear medicine evaluation in diagnostically ambiguous polyarthralgia.

## Data Availability

All clinical, laboratory, and imaging data that support the findings of this case report are available from the corresponding author (Roshan Karna; roshankarna1@gmail.com) upon reasonable request. All data have been fully anonymized in accordance with the patient’s written informed consent. Data sharing is subject to applicable institutional and ethical guidelines.

## Research registration unique identifying number (UIN)

Not applicable. This is a single case report and does not constitute a prospective clinical trial, interventional study, or systematic review requiring registration in a publicly accessible database. No research registration number is applicable.

## References

[1] Chan KL Mok CC. Glucocorticoid-induced avascular bone necrosis: diagnosis and management. Open Orthop J 2012;6:449–457. 10.2174/1874325001206010449 PMC3480825 23115605

[2] Agha R Mathew G Rashid R. Transparency in the reporting of Artificial INtelligence – the TITAN guideline. Prem J Sci 2025. doi:10.70389/PJS.100082

[3] Lee JW Chang SH Jang SJ. Clinical utility of quantitative analysis of bone scintigraphy in detecting clinically active joint and high disease activity in patients with rheumatoid arthritis. BMC Med Imaging 2021;21:177. 10.1186/s12880-021-00712-2 PMC8611961 34814863

[4] Wenger M Schirmer M. Indications for diagnostic use of nuclear medicine in rheumatology: a mini-review. Front Med (Lausanne) 2022;9:1026060. 10.3389/fmed.2022.1026060 PMC9554140 36250088

[5] Al-Nahhas A Zerizer I Jawad AS. The expanding role of nuclear medicine in rheumatology. Arab J Rheumatol 2023;1:31–35.

[6] Schmidkonz C Kuwert T Götz TI. Recent advances in nuclear medicine and their role in inflammatory arthritis: focus on the emerging role of FAPI PET/CT. Skeletal Radiol 2025;54:2243–2252. 10.1007/s00256-024-04834-w PMC12460390 39586916

[7] SequeiraSB JonesLC GoodmanSB MontMA. Associated risk factors, pathogenesis, diagnosis, and treatment options for multifocal osteonecrosis: a systematic review. J Arthroplasty 2025;40:S34–S40.10.1016/j.arth.2025.05.11040480326

